# An On‐Chip Second‐Order Elastic Topological Insulator for Demultiplexing Out‐of‐Plane and In‐Plane Corner Modes

**DOI:** 10.1002/advs.202411398

**Published:** 2024-12-04

**Authors:** Yafeng Chen, Lei Fan, Jie Zhu, Zhongqing Su

**Affiliations:** ^1^ Department of Mechanical Engineering The Hong Kong Polytechnic University Kowloon Hong Kong SAR 999077 China; ^2^ Institute of Acoustics School of Physics Science and Engineering Tongji University Shanghai 200092 China

**Keywords:** elastic metamaterials, elastic topological insulator, elastic waves, micro elastic devices

## Abstract

Second‐order elastic topological insulators (SETIs) with tightly localized corner states present a promising avenue for manipulating elastic waves in lower dimensions. However, existing SETIs typically support corner states of only a single mode, either out‐of‐plane or in‐plane. In this work, an on‐chip SETI that simultaneously hosts both high‐frequency out‐of‐plane and in‐plane corner states at ≈0.2 MHz is introduced. The presence of these corner states is experimentally validated, and their selective excitation by tuning the excitation frequency is demonstrated. This capability to demultiplex out‐of‐plane and in‐plane corner states positions the SETI as a potential platform for developing multifunctional elastic devices and enhancing the communication capacities of elastic waves. Furthermore, due to its structural simplicity, the SETI can be readily scaled and integrated into on‐chip elastic circuits, making it suitable for applications in micro‐electromechanical systems.

## Graphical abstract

An on‐chip second‐order elastic topological insulator (SETI) is developed, hosting high‐frequency out‐of‐plane and in‐plane corner states ≈0.2 MHz simultaneously. Experimental validation confirms the presence and selective excitation of these corner states by tuning the frequency. This SETI offers a promising platform for developing multifunctional micro‐electromechanical systems, enhancing the communication capacities of elastic waves.

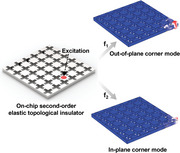

## 1 Introduction

Elastic topological insulators (ETIs) with topologically protected states offer an efficient route for steering elastic waves with robustness.^[^ ^1^, ^2^ ^]^ By drawing inspirations from topological insulators (TIs) in electronic, photonic, and acoustic systems, a variety of ETIs have been developed.^[^ ^2^ ^]^ Following the traditional bulk‐boundary correspondence, first‐order ETIs based on quantum Hall effects and quantum spin/valley Hall effects have been created, where *N*‐dimensional systems host (*N*‐1)‐dimensional edge states.^[^ ^3^, ^4^, ^5^, ^6^, ^7^, ^8^ ^]^ For applications in micro‐electromechanical systems (MEMS) aimed at the integrated communication of ultrasonic elastic waves, on‐chip ETIs that support high‐frequency (greater than 100 kHz) topological edge states have been developed.^[^ ^9^, ^10^, ^11^, ^12^, ^13^ ^]^ Extending beyond traditional bulk‐boundary correspondence, higher‐order ETIs that host multidimensional boundary states have also been proposed.^[^ ^14^ ^]^ For instance, a second‐order ETI (SETI) supports 1D gapped edge states and 0D in‐gap corner states. Given the crucial role of crystalline symmetry in determining the physical mechanisms of SETIs, various types of SETIs have been constructed in different symmetric lattices, including kagome, glide, C_6v_, C_4v_, C_3_, and so on.^[^ ^14^, ^15^, ^16^, ^17^ ^]^ Additionally, on‐chip SETIs with high‐frequency topological corner states have been developed for MEMS applications.^[^ ^18^ ^]^

Despite significant advancements in the development of SETIs, current SETIs typically host corner states of only one polarization, either out‐of‐plane (flexural) mode^[^ ^14^, ^15^, ^16^, ^17^, ^18^, ^19^, ^20^ ^]^ or in‐plane (coupled longitudinal and transverse) mode,^[^ ^21^ ^]^ as they only host nontrivial bandgap of one mode. This leaves the exploration of SETIs supporting both out‐of‐plane and in‐plane corner modes uncharted. For integrated communications of elastic waves, especially for on‐chip applications, it is highly desirable to design a system that hosts out‐of‐plane and in‐plane modes simultaneously, as this allows encoding different information into different modes, thereby multiplying the communication capacities of the elastic circuit.^[^ ^22^, ^23^ ^]^ Meanwhile, as the out‐of‐plane and in‐plane modes of elastic waves have different functions, the SETI that supports both modes can be also used for designing multifunctional elastic devices.

Here, we develop an on‐chip SETI that hosts both out‐of‐plane and in‐plane corner states at high frequencies (≈0.2 MHz). We experimentally demonstrate that these corner states can be selectively excited by tuning the frequency of the same excitation source. This SETI has the potential to enhance communication capacities of on‐chip elastic devices. Furthermore, with the ability to demultiplex in‐plane and out‐of‐plane corner modes, the SETI can be employed to develop multifunctional MEMS by leveraging the unique functions of each specific mode.

## 2 Results

**Figure 1:**
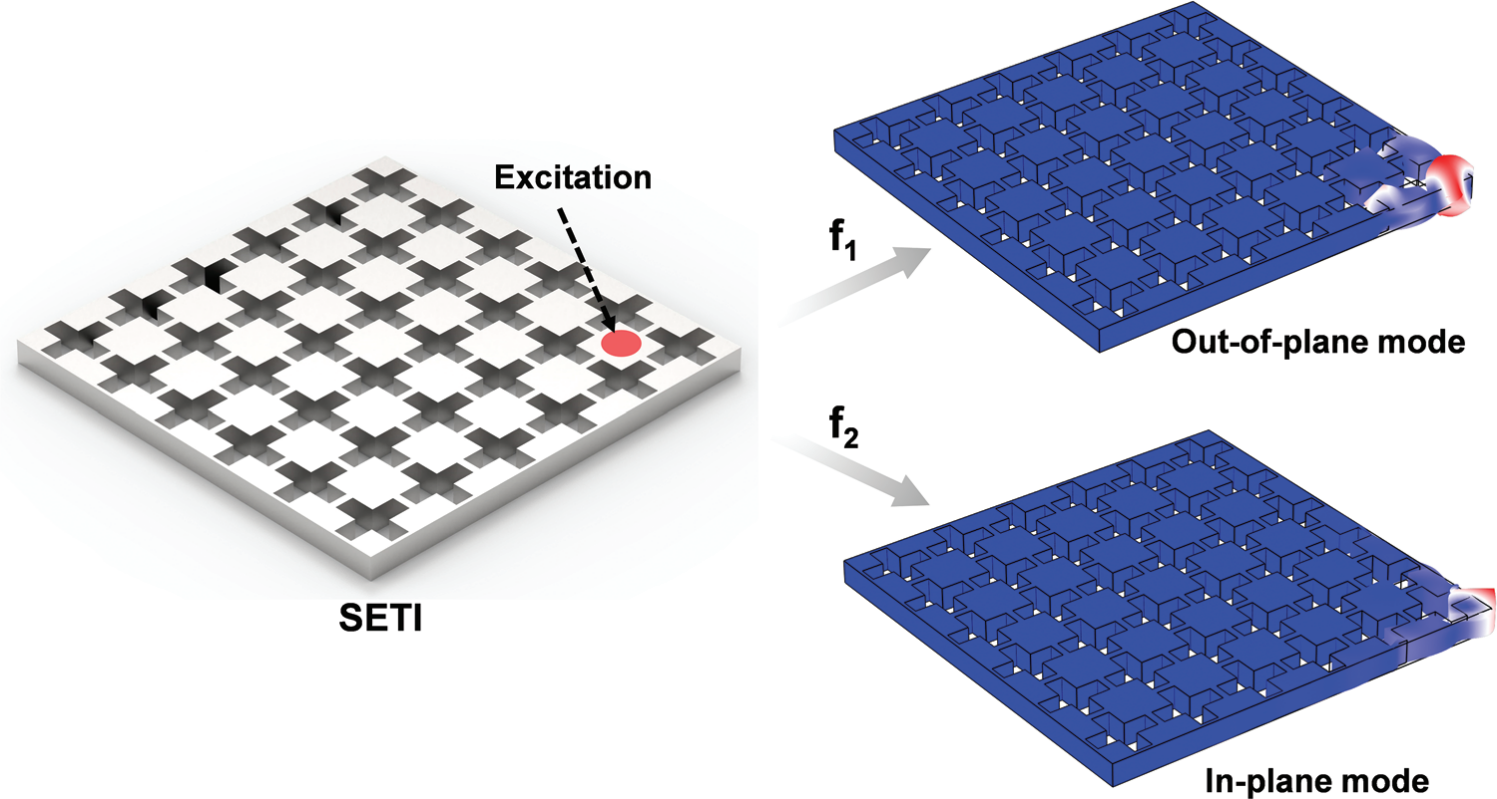
Schematic of the developed SETI for demultiplexing out‐of‐plane and in‐plane corner states.

**Figure 2:**
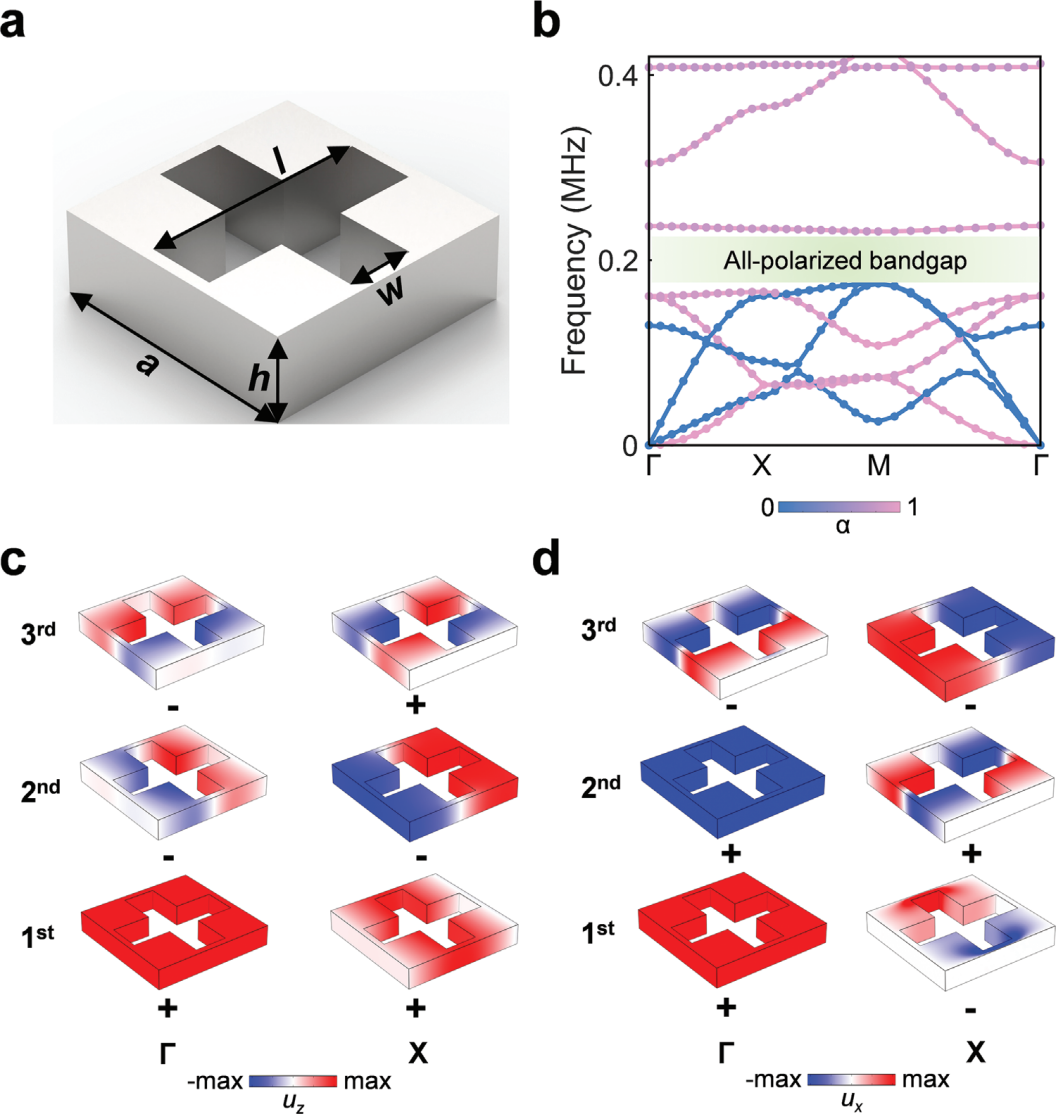
The sketch of the UC, its band diagram and the eigenmodes of each band at high‐symmetric points. a) The sketch of the UC. b) The band diagram of the UC. c,d) Eigenmodes at Г and *X* points of 1st–3rd bands for (c) the out‐of‐plane mode and (d) the in‐plane mode.

**Figure** **1** illustrates the schematic of the developed SETI designed for demultiplexing out‐of‐plane and in‐plane corner states. This SETI supports corner states for both out‐of‐plane and in‐plane modes with distinct frequencies. By adjusting the frequency of the excitation source, these corner states can be selectively excited, enabling various applications. **Figure** **2**a depicts the sketch of the unit cell (UC), which is made of an aluminium plate perforated with a cross‐shaped hole. The detailed geometric parameters are as follows: *a* = 6 mm, *h* = 2 mm, *l* = 5.4 mm and *w* = 1.6 mm. Here, we employ two methods to calculate the band diagram of the UC. In the first method, we directly compute the all‐polarized eigenmodes corresponding to the wave vectors along the boundary of the first Brillouin zone [Γ(0,0)‐*X*(π/*a*,0)‐*M*(π/*a*,π/*a*)‐Γ(0,0)], as indicated by the scatter points in Figure 2b. We then characterize the polarization of the eigenmodes using a polarization factor defined by $\alpha =\int \int \int_{UC}\frac{{|u_{z}|}^{2}}{\left({|u_{x}|}^{2}+{|u_{y}|}^{2}+{|u_{z}|}^{2}\right)}dV\left(0\leq \alpha \leq 1\right)$,^[^ ^24^ ^]^ where *u_x_*, *u_y_* and *u_z_* mean the *x−*, *y−*, and *z−*polarized displacement component of the eigenmode, respectively, and *V* represents the volume of the UC. α = 1 means the out‐of‐plane mode while α = 0 indicates the in‐plane mode. In the second method, we independently compute the out‐of‐plane and in‐plane modes using the methodology described in Refs.,^[^ ^25^, ^26^ ^]^ with the results depicted by pink and blue lines in Figure 2b, respectively. We can find that the band dispersions of both out‐of‐plane and in‐plane modes calculated by these two methods are consistent, indicating that the all‐polarized modes within this system can be decoupled into out‐of‐plane and in‐plane modes effectively. In Figure 2b, we observe an all‐polarized bandgap ranging from 0.175 to 0.231 MHz, with a relative bandwidth of 27.6%. Note that engineering such an all‐polarized bandgap is also a prerequisite for the emergence of both out‐of‐plane and in‐plane corner states. In (Section S1, Supporting Information), we also provide a topology optimization method to maximize the all‐polarized bandgap to host more localized corner states. We then analyze the topological properties of the UC for out‐of‐plane and in‐plane modes, respectively, which can be characterized by the 2D polarization **P** = (*P_x_*, *P_y_*),^[^ ^19^, ^24^, ^27^, ^28^, ^29^, ^30^, ^31^ ^]^ where

(1) $$P_{j}=\frac{1}{2}\left(\sum_{l}q_{j}^{l}modulo2\right),\;\;\;{\left(-1\right)}^{q_{j}^{l}}=\frac{\eta_{l}\left(X_{j}\right)}{\eta_{l}\left(\Gamma \right)}$$

where *j* represents the *x−* or *y−*direction and as the UC has the C_4v_ point group symmetry, *P_x_* = *P_y_*. η_*l*_(*X_j_*) and η_*l*_(Γ) denote the parity (±) of the *l^th^* band at high symmetry points *X* and Γ, respectively. The parities are determined by the field profiles of the corresponding eigenmodes, that is, the monopolar and quadrupolar modes have an even parity (+), whereas the dipolar mode possesses an odd parity (−). The $\sum_{l}q_{j}^{l}$ means the summation of *q* over all bands below the bandgap. Figure 2c,d show the eigenmodes at Г and *X* points of bands below the bandgap for the out‐of‐plane mode and the in‐plane mode, respectively. Note that the *u_y_*‐polarized eigenmodes exhibit the same parity with the *u_x_*‐polarized eigenmodes at each high‐symmetric point for the in‐plane mode. Consequently, we derive that **P** = (1/2, 1/2) for the UC for both out‐of‐plane and in‐plane modes, indicating that the UC is topologically nontrivial for both modes. Meanwhile, we further calculate the topological index of the UC, formulated asχ = ([*X*_1_],[*Y*_1_],[*M*_1_]), where $\left[\Pi_{1}\right]=\#\Pi_{1}-\#\Gamma_{1}\left(\Pi =X,Y,M\right)$ with $\#\Pi_{1}$ and $\#\Gamma_{1}$ denoting the number of eigenmodes with even parity at Π and Γ below the bandgap, respectively.^[^ ^18^ ^]^ As the UC is C_4v_‐symmetric, [*X*_1_] = [*Y*_1_] for them. **Table** **1** and **Table** **2** present parities at high‐symmetric points of all bands below the bandgap for the UC in out‐of‐plane and in‐plane modes, respectively. We can derive that χ = (1, 1, 0) and χ = (−1, −1, 0) for the UC in out‐of‐plane and in‐plane modes, respectively. Such nonzero topological indexes under the symmetry analysis further verify that UC is topological nontrivial for both out‐of‐plane and in‐plane modes. Based on the χ, we can further calculate the corner charge, $Q_{c}=-\frac{1}{4}\left(\left[X_{1}\right]+\left[Y_{1}\right]-\left[M_{1}\right]\right)$, that characterizes the high‐order topology. We can derive that $Q_{c}=-\frac{1}{2}$ and $Q_{c}=\frac{1}{2}$ for the UC in out‐of‐plane and in‐plane modes, respectively. The nonzero corner charges enable the construction of a SETI by arranging the UC, capable of hosting corner states for both out‐of‐plane and in‐plane modes.

**Table 1:** Parities at high‐symmetric points of 1st–3rd bands for the UC in the out‐of‐plane mode.

	Band order		
	1	2	3
Г	+	−	−
*X*	+	−	+
*M*	−	−	+

**Table 2:** Parities at high‐symmetric points of 1st–3rd bands for the UC in the in‐plane mode.

	Band order		
	1	2	3
Г	+	+	−
*X*	−	+	−
*M*	−	+	+

**Figure 3:**
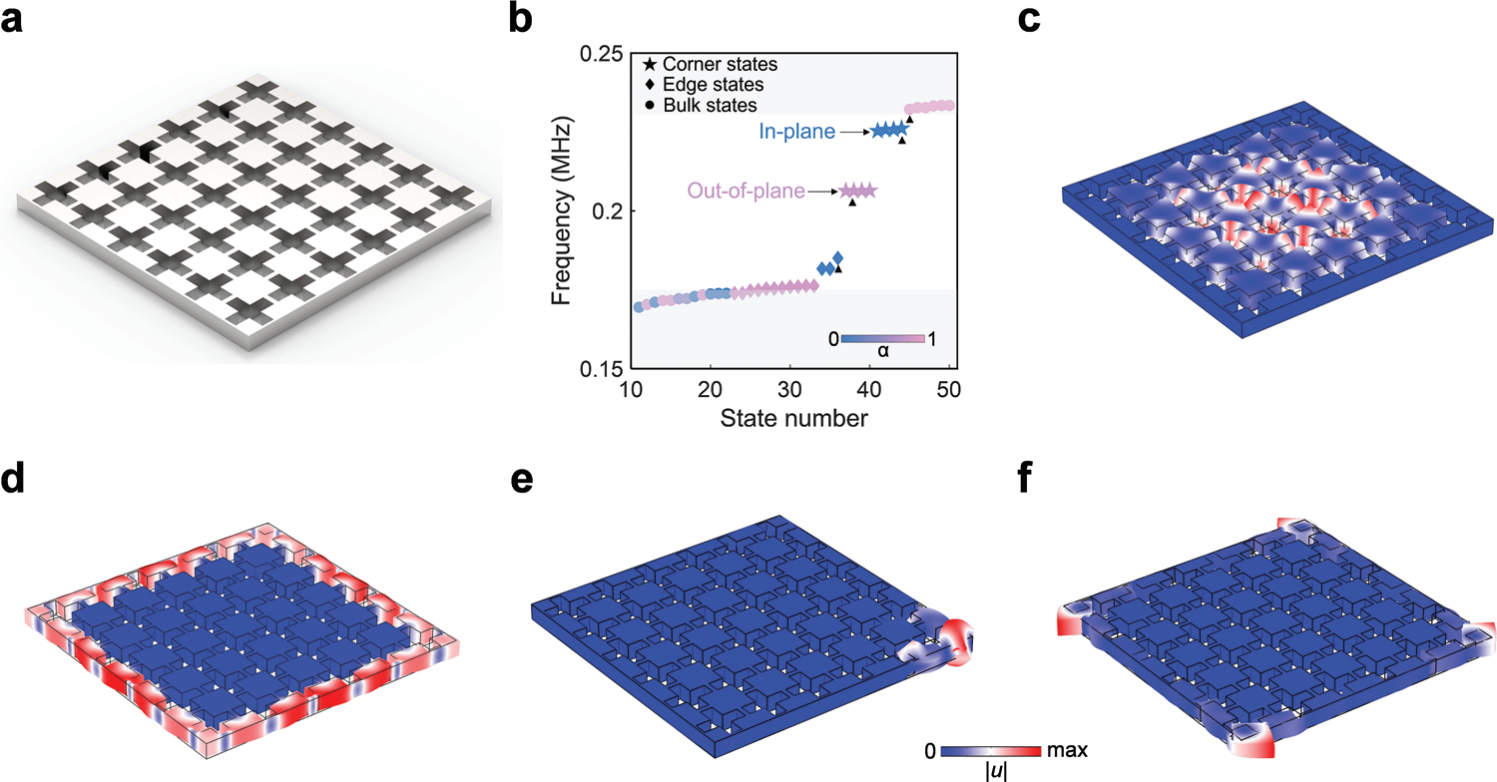
The numerical calculation of corner states. a) Sketch of the SETI. b) Eigenfrequency spectrum of the SETI. c) The representative eigenmode of the bulk state. d) The representative eigenmode of the edge state (in‐plane). e) The representative eigenmode of the out‐of‐plane corner state. f) The representative eigenmode of the in‐plane corner state. The representative eigenmodes of bulk, edge, and corner states are denoted by the black triangles in (b).

To demonstrate the corner states, we construct the SETI composed of a 6 × 6 array of UCs, as illustrated in **Figure** **3**a, and calculate its eigenfrequency spectrum, as shown in Figure 3b. Edge states and two groups of corner states emerge within the all‐polarized bandgap; each group consists of four degenerated corner states. Figure 3c,d demonstrate the representative eigenfields of the bulk and edge states, showing that energies are distributed within the bulk and along the boundary, respectively. Figure 3e,f display the representative eigenfields of these two groups of corner states, with the frequency being 0.207 and 0.226 MHz, respectively. The eigenfields of the other three corner states of each group are given in (Section S2, Supporting Information). We can find that the energies are highly localized at the corners. The calculated polarization factors for the corner states in Figure 3e,f are 0.634 and 0.002, indicating that the vibration modes of these two corner states are dominated by out‐of‐plane and in‐plane motions, respectively. Meanwhile, in (Section S3, Supporting Information), we also demonstrate that the corner states still exist when we replace the aluminum with steel or silicon, demonstrating the material flexibility of the designed SETI.

**Figure 4:**
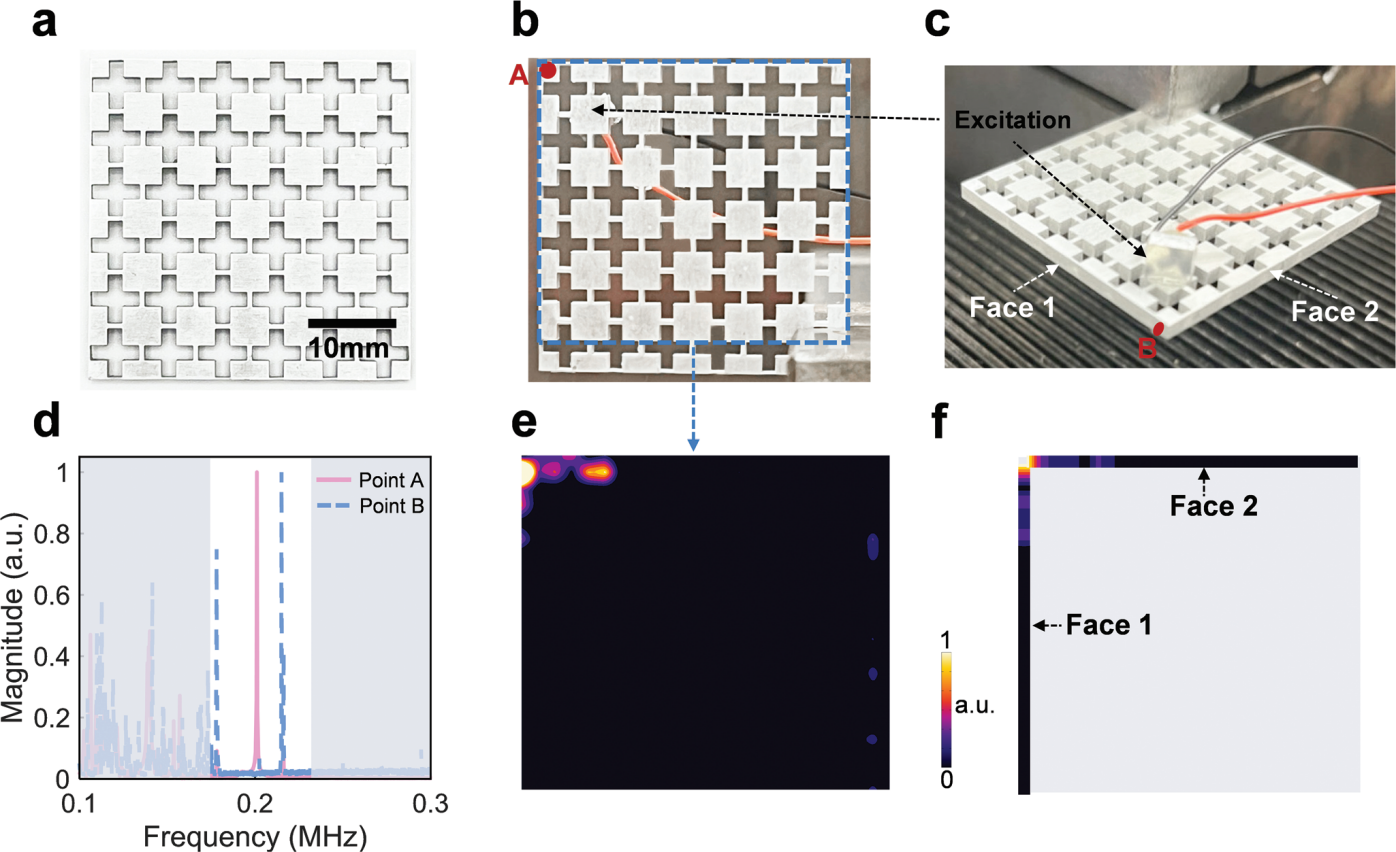
The experimental validation of corner states. a) The fabricated SETI. b,c) The schematic of scanning faces for measuring (b) out‐of‐plane and (c) in‐plane vibration responses, respectively. d) The response spectrum of point A and point B denoted by the red circles in (b) and (c). e,f) The scanned (e) out‐of‐plane vibration responses at 0.201 MHz and (f) in‐plane vibration responses at 0.215 MHz.

To experimentally validate the corner states, we fabricate the SETI by perforating an aluminum plate (6061‐aluminum alloy) using laser cutting technology, as shown in **Figures** **4**a. The excitation source is generated via a piezoelectric disk (PZT‐5H, 5 mm diameter, 1 mm thickness) attached to an aluminum patch (dimensions: 5 mm × 5 mm × 2 mm) affixed to the surface of the sample, as indicated in Figure 4b,c. Similar to ref. [18] we put the excitation source near to the corner so that the corner states can be well excited. This setup allows for the excitation of both out‐of‐plane and in‐plane modes. The vibration responses of the sample are captured using a scanning laser vibrometer (Polytec, PSV‐500). Since the laser vibrometer can only measure vibrational responses perpendicular to the scanned face, we scan the front face (denoted by the blue dashed lines in Figure 4b) to validate the out‐of‐plane corner states, and the two side faces (denoted as Face 1 and Face 2 in Figure 4c) to confirm the in‐plane corner states. Figure 4d shows the response spectrum at points A and B at the corner of the sample, as denoted by the red circles in Figure 4b,c. Response peaks appear within the frequency window of the all‐polarized bandgap (between the gray areas) for both points A and B, with frequencies of 0.201 and 0.215 MHz, respectively. From the scanned field distributions shown in Figure 4e,f corresponding to these two peaks, we observe that the out‐of‐plane vibration responses at 0.201 MHz and in‐plane vibration responses at 0.215 MHz are highly localized at the corner, confirming the coexistence of out‐of‐plane and in‐plane corner states experimentally. Furthermore, the experiment result demonstrates that out‐of‐plane and in‐plane corner states can be selectively excited by tuning the frequency, which is mainly due to the fact that they have different eigenfrequencies. This indicates that the developed SETI is capable of converting all‐polarized elastic waves into different modes at different frequencies. The relative errors in the frequencies of the out‐of‐plane and in‐plane corner states between experiment and simulation are 2.9% and 4.9%, respectively, which are primarily due to manufacturing‐induced geometry errors, such as variations in the width of the slender rods connecting neighboring blocks. Such errors are within acceptable experimental error ranges.^[^ ^18^ ^]^ Note that, within the bandgap, we also observe an extra peak of the in‐plane vibration responses, which corresponds to the edge states, as indicated by the blue rhombus in Figure 3b. Meanwhile, there are almost no responses above the all‐polarized bandgap in Figure 4d. This is due to the fact that there is only a narrow bulk band, with the frequency window of (0.231, 0.237 MHz), between 0.2 and 0.3 MHz, as can be seen in Figure 2b. However, from the eigenmodes of the bulk states of this band for the SETI, presented in (Section S4, Supporting Information), we can find that the displacements at the corners and along the boundaries are almost zero. Therefore, there are no obvious responses within this frequency window of the response spectrum captured at the corner even though these bulk states are excited.

## 3 Conclusion

To conclude, we have developed an on‐chip SETI that simultaneously supports high‐frequency out‐of‐plane and in‐plane corner states, which can be seamlessly integrated into on‐chip elastic circuits for MEMS applications. We experimentally demonstrate that these corner states can be selectively excited by tuning the excitation frequency. The developed SETI exhibits significant potential across a range of applications. By hosting both out‐of‐plane and in‐plane corner states with tightly localized energies, it can be utilized for harvesting ultrasonic energies from all‐polarized elastic waves,^[^ ^23^ ^]^ which can subsequently inform the design of self‐powered on‐chip elastic devices. Additionally, its ability to demultiplex these corner modes positions the SETI as a versatile tool for creating multifunctional elastic devices capable of performing diverse tasks, such as defect detection using distinct modes. Furthermore, the SETI can function as a mode splitter in elastic circuits, transforming all‐polarized elastic waves into specific modes, thereby enhancing communication capacities by encoding different information into separate modes. With advancements in nanolithography, the SETI can be further miniaturized to manipulate GHz elastic waves, paving the way for innovative applications in elastic wave technology.^[^ ^32^ ^]^

## 4 Experimental Section

### Numerical Simulation

The COMSOL Multiphysics Solid Mechanics Module was adopted for calculating the band diagram of the UC and eigenfrequency spectrum of the SETI. For calculating the band diagram of the UC, two methods were adopted. In the first method, the four boundaries parallel to the *z*‐axis were set as Floquet periodic boundary conditions. Then the eigenfrequencies of the full UC were calculated by sweeping wave vectors along the first Brillouin zone boundary, as shown by the colourful points in Figure 2b. In the second method, the thickness of the UC was set as *t*/2 and the four boundaries parallel to the *z*‐axis were set as Floquet periodic boundary conditions. Then, the *z*‐polarized displacement of the bottom boundary was constrained to be zero to get the eigenfrequencies of the in‐plane mode, and the *x*− and *y*−polarized displacements were constrained to be zero to get the eigenfrequencies of the out‐of‐plane modes. For calculating the eigenfrequency spectrum of the SETI, all boundaries were set as free boundary conditions and the eigenfrequencies were directly calculated. The material of the SETI is aluminum with the elastic modulus, density, and Poisson ratio of 70 GPa, 2700 kg m^−3^, and 0.33, respectively. In (Section S5, Supporting Information), the effects of the excitation positions on the response of the corner states were also discussed.

### Experimental Setup

**Figure 5:**
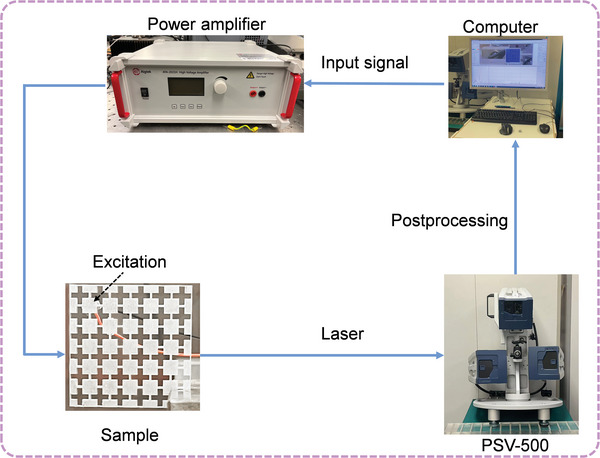
The signal flow of the experimental devices.

The sample was manufactured by perforating an aluminum plate (6061‐aluminum alloy), with the length, width, and thickness of 36, 36 and 2 mm, respectively, via the laser cutting technology with a precision of 0.2 µm. The excitation source was generated via a piezoelectric disk (PZT‐5H, 5 mm diameter, 1 mm thickness) attached to an aluminum patch (dimensions: 5 mm × 5 mm × 2 mm) affixed to the surface of the sample for the excitation of both out‐of‐plane and in‐plane modes. The piezoelectric disk is actuated by a 0.01–1 MHz up‐chirp signal emitted by a computer and amplified by a power amplifier (Aigtek, ATA‐2021H). The vibration responses of the sample were captured using a scanning laser vibrometer (Polytec, PSV‐500). **Figure** **5** presents the signal flow of the experimental devices.

## Conflict of Interest

The authors declare no conflict of interest.

## Supporting information



Supporting Information

## Data Availability

The data that support the findings of this study are available from the corresponding author upon reasonable request.
