# Supplementary material for: An On‐Chip Second‐Order Elastic Topological Insulator for Demultiplexing Out‐of‐Plane and In‐Plane Corner Modes
Source: Adv Sci (Weinh). 2024 Dec 4;12(4):2411398. doi: 10.1002/advs.202411398 (PMC11775516; doi:10.1002/advs.202411398)
Supplement: Supplementary file 1 — Supporting Information [file ADVS-12-2411398-s001.docx]

# Susspplementary Materials for “An On-chip Second-order Elastic Topological Insulator for Demultiplexing Out-of-plane and In-plane Corner Modes”

Yafeng Chen^1#^, Lei Fan^1^, Jie Zhu^2⸾^, and Zhongqing Su1[[1]](#footnote-1)*

^1^*Department of Mechanical Engineering, The Hong Kong Polytechnic University, Kowloon, Hong Kong SAR, China*

^2^*Institute of Acoustics, School of Physics Science and Engineering, Tongji University, 200092 Shanghai, China*

# 1. The topology optimization method for maximizing the all-polarized band gap

As the width of the all-polarized bandgap can determine the localization degree (quality factor) of the out-of-plane and in-plane corner states, we here present the topology optimization method for maximizing the all-polarized bandgap. For elastic waves propagating within the plate structure with linear isotropic material properties, the governing equation can be expressed as

(S1)

where $\text{U=}\left[u_{x},u_{y},u_{z}\right]$ denotes the displacement vector. $\lambda$ and $\mu$ denote the elastic Lame constants. $\rho$ represents the density and is the gradient operator. Due to the periodicity of the phononic crystal (PC), $\rho\left(r\right)=\rho\left(r+R\right)$, $\lambda\left(r\right)=\lambda\left(r+R\right)$ $\mu\left(r\right)=\mu\left(r+R\right)$, where **r** and **R** denote the position vector and lattice translation vector, respectively. Following the Bloch-Floquet theorem [1],

$\text{U}\left(r,k\right)=\text{U}_{k}\left(r\right)e^{i\left(\omega t+k\cdot r\right)}$ (S2)

where $k=\left(k_{x},k_{y}\right)$ denotes the wave vector. *ω* represents the angular frequency. $\text{U}_{k}\left(r\right)\text{=U}_{k}\left(r\text{+}R\right)$ denotes the periodic displacement function, which has the same periodicity as the PC. Taking Eq. (S2) into Eq. (S1) and using the finite element method, we can get the eigenvalue equation of Eq. (S1). Via setting the thickness of the unit cell as half of the original thickness and constraining the *z*-polarized displacement (*x*- and *y*-polarized displacement) of the bottom boundary to be zero, we can get the eigenvalue equation of the in-plane (out-of-plane) mode,

$\left(K_{in(out)}-\omega_{in(out)}^{2}M_{in(out)}\right)U_{in(out)}=0$ (S3)

where $K$ and $M$ denote the global stiffness and mass matrix, respectively. **U** denotes the eigenvector of the displacement field. The subscript *in* and *out* represent in-plane and out-of-plane modes, respectively. By sweeping the wave vector **k** along the boundary of the first Brillouin zone, we can get the band diagrams of in-plane and out-of-plane modes, respectively. Here, the relative size of the all-polarized bandgap is selected as the optimization objective, expressed by,

$g_{n}=2\frac{\min_{i=1,2,\cdots n_{k}} \omega_{n+1}\left(k_{i}\right)-\max_{i=1,2,\cdots n_{k}} \omega_{n}\left(k_{i}\right)}{\min_{i=1,2,\cdots n_{k}} \omega_{n+1}\left(k_{i}\right)+\max_{i=1,2,\cdots n_{k}} \omega_{n}\left(k_{i}\right)}$ (S4)

where $n_{k}$ denotes the total number of wave vectors considered. *n* denotes the total bands, including both in-plane and out-of-plane modes, below the bandgap after combining the bands of these two modes. To avoid numerical instabilities, we adopt the strategy proposed in Refs. [2,3] to modify the objective function. We assign each element a design variable $x_{e}$, where $x_{e}=1$ means the element is aluminum and $x_{e}=0$ denotes the element is air. For the element with intermediate value, we adopt the linear material interpolation to represent its density and elastic Lame constants. Then, the sensitivity of the objective function, $\frac{\partial g_{n}}{\partial x_{e}}$, can be derived upon getting $\frac{\partial\omega_{n}\left(k_{i}\right)}{\partial x_{e}}$, which can be calculated by differentiating both sides of Eq. (S3),

$\frac{\partial\omega_{n}\left(k_{i}\right)}{\partial x_{e}}=\frac{1}{2\omega_{n}}U^{T}\left(\frac{\partial K}{\partial x_{e}}-\omega_{n}^{2}\left(k_{i}\right)\frac{\partial M}{\partial x_{e}}\right)U$ (S5)

After getting the sensitivity of each element, we can use the bi-directional evolutionary optimization method [3] to update the design variable of each element iteratively until the objective function is maximized. Note that, according to previous studies [4], we should set the order of the bandgap as an odd value for both out-of-plane and in-plane modes in order to get a nontrivial bandgap.

# 2. Eigenmodes of out-of-plane and in-plane corner modes

From the **Figure 3b** in the main text, we can find that each group of corner states include four degenerated eigenmodes. Apart from the representative eigenmodes of out-of-plane and in-plane corner states in **Figure 3** of the main text, we also give the other three eigenmodes of out-of-plane (in-plane) corner states in **Figures S1a- S1c** (**Figures S1d- S1f**), respectively.


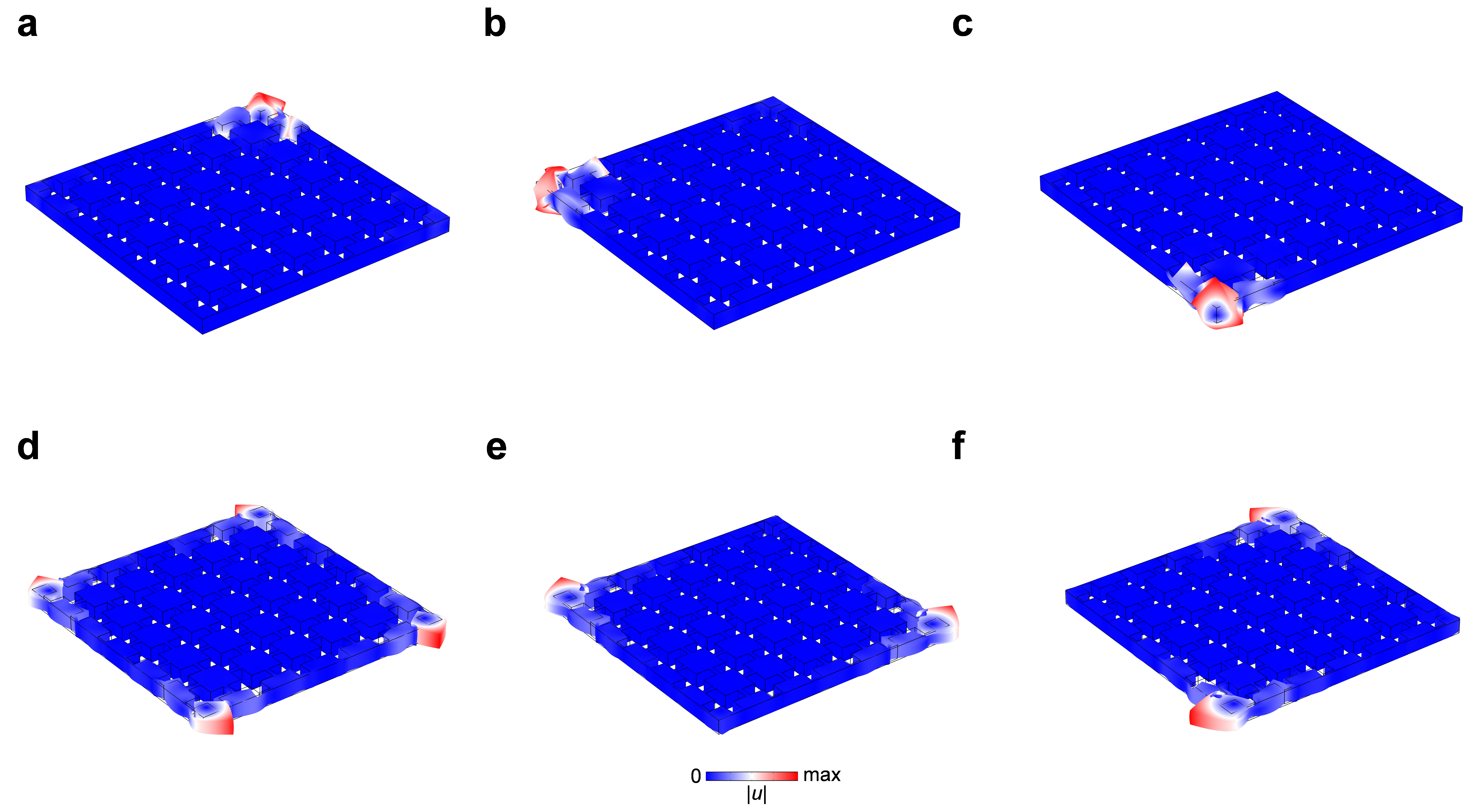


**Figure S1**. (a-c) The other three eigenmodes of out-of-plane corner states. (d-f) The other three eigenmodes of in-plane corner states.

# 3. The material flexibility of the second-order elastic topological insulator (SETI)

To demonstrate the material flexibility of the developed structure, we replace the aluminium with silicon or steel and then calculate the eigenfrequency spectrum of the SETI. The adopted elastic modulus, density and Poisson ratio of silicon are 130 GPa, 2230 kg/m^3^ and 0.27, while they are 200 GPa, 7850 kg/m^3^ and 0.3 for steel, respectively. **Figure S2a** (**Figure S2d**) shows the eigenfrequency spectrum of the SETI made of silicon (steel), demonstrating that both out-of-plane and in-plane corner states still appear within the bandgap. **Figure S2b** (**Figure S2e**) and **Figure S2c** (**Figure S2f**) present the eigenmodes of out-of-plane and in-plane corner states for the SETI made of silicon (steel), respectively. Therefore, the developed structure possesses material flexibility.


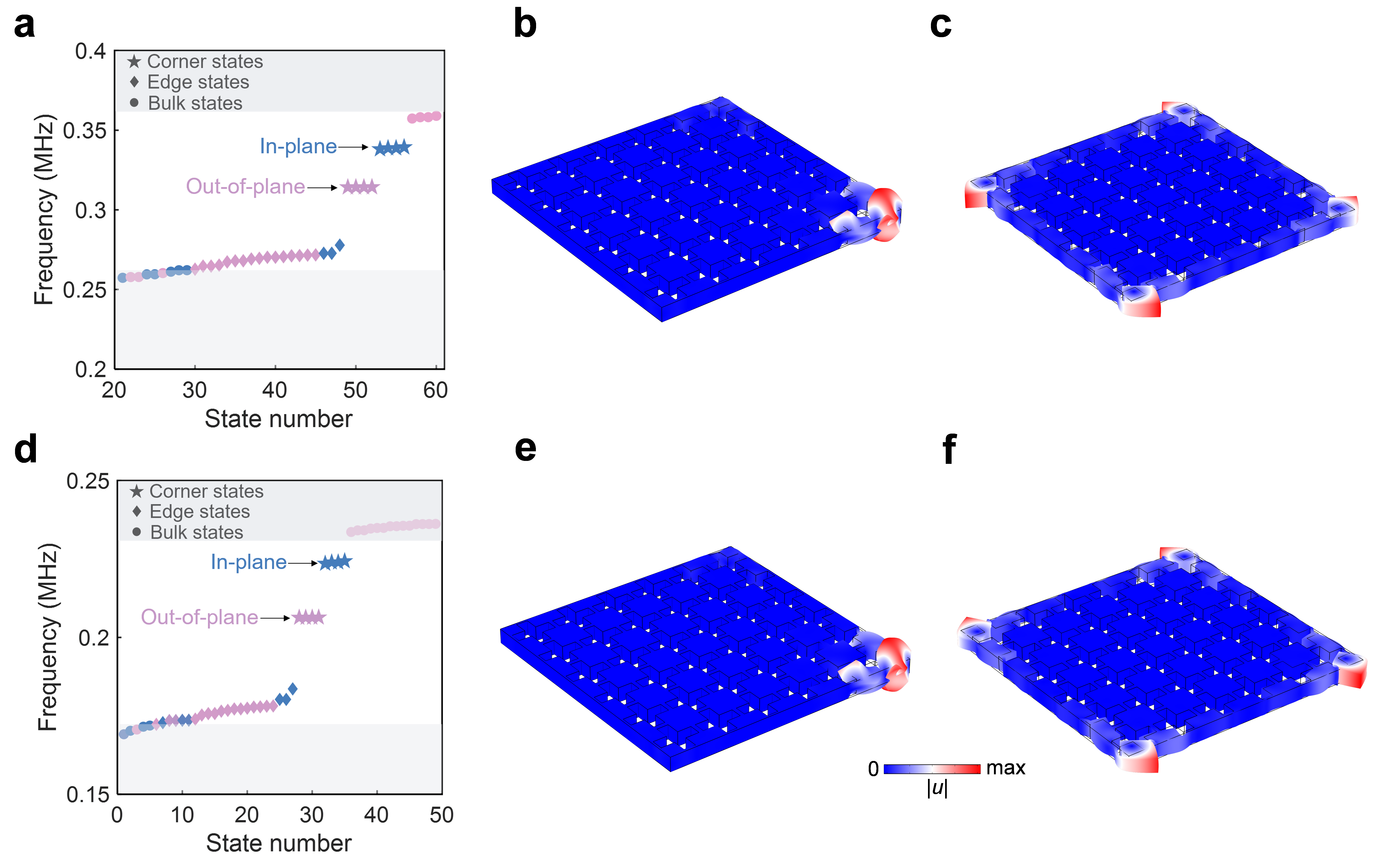


**Figure S2.** The numerical calculation of corner states of the SETI made of silicon and steel. (a-c) The eigenfrequency spectrum of the SETI made of silicon, the eigenmode of the out-of-plane corner state and eigenmode of the in-plane corner state, respectively. (d-f) The eigenfrequency spectrum of the SETI made of steel, the eigenmode of the out-of-plane corner state and eigenmode of the in-plane corner state, respectively.

# 4. The eigenmodes of the bulk states of the narrow band above the all-polarized bandgap

**Figure S3** shows the calculated eigenmodes of the bulk states of the narrow band above the all-polarized bandgap for the SETI. We can find that the displacements at the corners and along the boundaries are almost zero. Therefore, there are no signals within this frequency window of the response spectrum captured at the corner, even though these bulk states are excited.


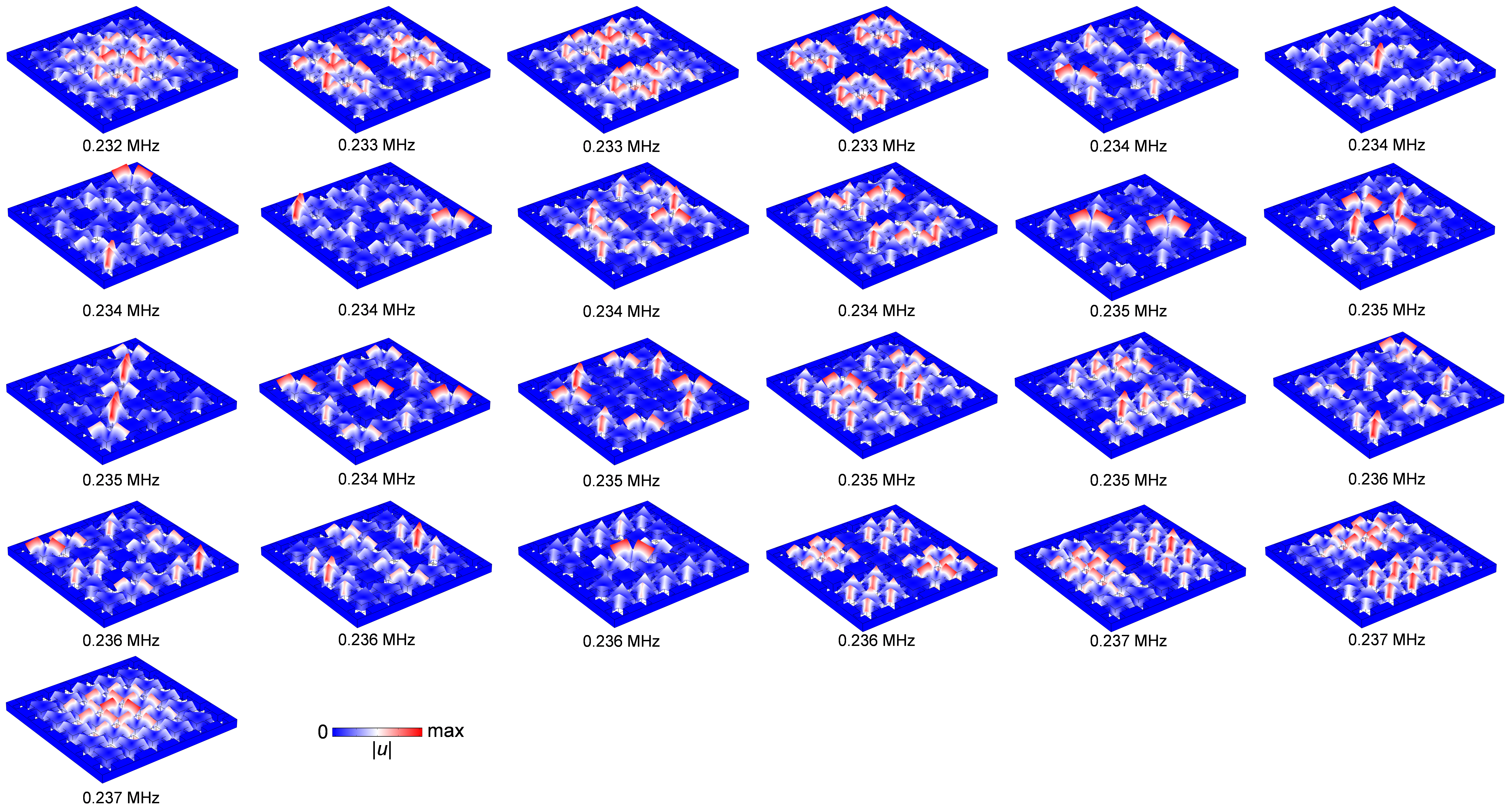


**Figure S3**. All eigenmodes of bulk states within the frequency window of (0.231 MHz, 0.237 MHz).

# 5. The effects of the excitation positions on the response of the corner states

To discuss the effects of the excitation positions on the response of the corner states, we place the source at various positions (denoted by B1, B2, E1, and E2 in **Figure S4a**, respectively) and capture the out-of-plane response (the absolute *z*-polarized displacement, |*u_z_*|) of the point A and in-plane response (the absolute *x*-polarized displacement, |*u_x_*|) of the point B. The points B1 and B2 are within the bulk while E1 and E2 locate at the side face. **Figure S4b (Figure S4c)** show the captured out-of-plane response of point A (in-plane response of point B) when the source is put at B1 and B2, respectively. **Figure S4d (Figure S4e)** show the captured out-of-plane response of point A (in-plane response of point B) when the source is put at E1 and E2, respectively. We can find that, corresponding to the eigenfrequencies of out-of-plane and in-plane corner states (0.207 MHz and 0.226 MHz, respectively), there are peaks of the response spectra, indicating that the corner states are excited. However, as the distance between the source and the corner increases, the response peaks significantly decrease. For example, the response peaks when the excitation is applied at points B1 and E1 are significantly higher than those captured when the excitation is applied at points B2 and E2. Note that the response peaks near the bottom boundary of the all-polarized bandgap indicate the excitation of edge states, which align well with the measured results in **Figure 4** of the main text.


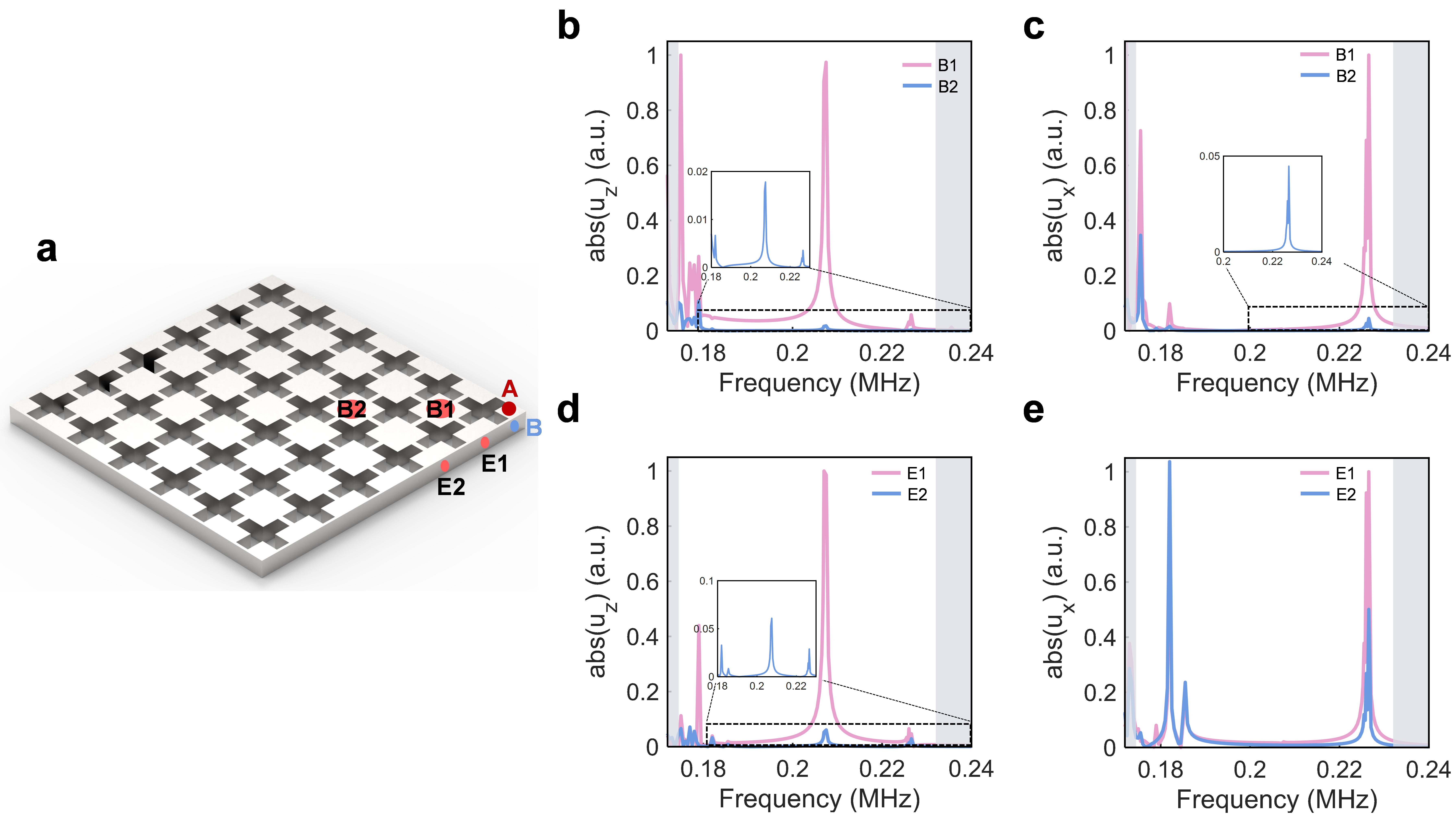


**Figure S4.** The response spectrum under different excitations. (a) Schematic of the excitation positions. (b) The out-of-plane response of point A when the source is put at points B1 and B2. (c) The in-plane response of point B when the source is put at points B1 and B2. (d) The out-of-plane response of point A when the source is put at points E1 and E2. (e) The in-plane response of point B when the source is put at points E1 and E2.

# 6. The eigenfrequency spectrum of a trivial structure

We construct a unit cell (named UCT) by moving the nontrivial unit cell in the main text with half the lattice size along both the horizontal and vertical directions, as shown in **Figure S5a**, and then construct a meta-structure with 6×6 UCTs, as shown in **Figure S5b**. **Table 1** and **Table 2** present parities at high-symmetric points of all bands below the bandgap for the UCT in out-of-plane and in-plane modes, respectively. We can derive that $\chi=\left(0,0,0\right)$ for the UCT in both out-of-plane and in-plane modes, meaning that UCT is trivial for these two modes. **Figure S5c** shows the eigenfrequency spectrum of the structure shown in **Figure S5b**, illustrating that there are not edge and corner states within the all-polarized bandgap, confirming that the nontrivial structure hosts topological corner states, while the trivial structure does not.


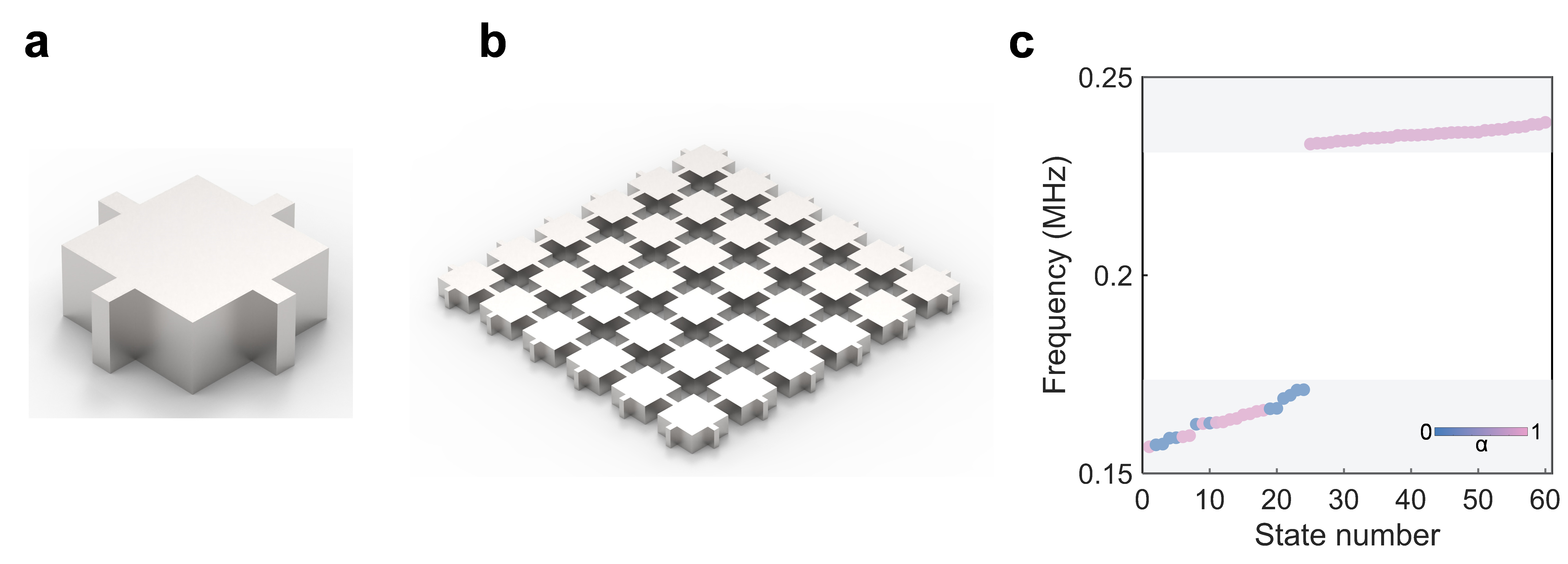


**Figure S5.** (a) The sketch of the UCT. (b) The sketch of the structure made of 6×6 UCTs. (c) The eigenfrequency spectrum of the structure in (b).

**Table S1.** Parities at high-symmetric points of 1^st^-3^rd^ bands for the UC in the out-of-plane mode.

|  | Band order | | |
| --- | --- | --- | --- |
|  | 1 | 2 | 3 |
| Г | + | - | - |
| *X* | - | + | - |
| *M* | - | - | + |

**Table S2.** Parities at high-symmetric points of 1^st^-3^rd^ bands for the UC in the in-plane mode.

|  | Band order | | |
| --- | --- | --- | --- |
|  | 1 | 2 | 3 |
| Г | + | + | - |
| *X* | + | - | + |
| *M* | - | + | + |

# Reference

[1] C. Kittel, Introduction to solid state physics : 6th ed, American Journal of Physics 61 (2011) 59.

[2] F. Meng, X. Huang, B. Jia, *J. Comput. Phys.* **2015**, 302, 393-404.

[3] Y.F. Li, X. Huang, F. Meng, S. Zhou, *Struct. Multidisc. Optim.* **2016**, 54, 595-617.

[4] Y. Chen, X. Wen, Z. Gu, J. Zhu, Z. Su, *Int. J. Mech. Sci.* **2023**, 260, 108669.

1. Corresponding authors:

   ^#^yachen@polyu.edu.hk.

   ^⸾^ [jiezhu@tongji.edu.cn](mailto:jiezhu@tongji.edu.cn)

   ^*^ [zhongqing.su@polyu.edu.hk](mailto:zhongqing.su@polyu.edu.hk) [↑](#footnote-ref-1)
